# An effective assay for high cellular resolution time-lapse imaging of sensory placode formation and morphogenesis

**DOI:** 10.1186/1471-2202-12-37

**Published:** 2011-05-09

**Authors:** Celia E Shiau, Raman M Das, Kate G Storey

**Affiliations:** 1 Neural Development Group, Division of Cell & Developmental Biology, College of Life Science, University of Dundee, Dundee DD1 5EH, Scotland, UK; 2 Current Address: Department of Developmental Biology, Stanford University School of Medicine, Stanford, CA 94305, USA

## Abstract

**Background:**

The vertebrate peripheral nervous system contains sensory neurons that arise from ectodermal placodes. Placodal cells ingress to move inside the head to form sensory neurons of the cranial ganglia. To date, however, the process of placodal cell ingression and underlying cellular behavior are poorly understood as studies have relied upon static analyses on fixed tissues. Visualizing placodal cell behavior requires an ability to distinguish the surface ectoderm from the underlying mesenchyme. This necessitates high resolution imaging along the z-plane which is difficult to accomplish in whole embryos. To address this issue, we have developed an imaging system using cranial slices that allows direct visualization of placode formation.

**Results:**

We demonstrate an effective imaging assay for capturing placode development at single cell resolution using chick embryonic tissue ex vivo. This provides the first time-lapse imaging of mitoses in the trigeminal placodal ectoderm, ingression, and intercellular contacts of placodal cells. Cell divisions with varied orientations were found in the placodal ectoderm all along the apical-basal axis. Placodal cells initially have short cytoplasmic processes during ingression as young neurons and mature over time to elaborate long axonal processes in the mesenchyme. Interestingly, the time-lapse imaging data reveal that these delaminating placodal neurons begin ingression early on from within the ectoderm, where they start to move and continue on to exit as individual or strings of neurons through common openings on the basal side of the epithelium. Furthermore, dynamic intercellular contacts are abundant among the delaminating placodal neurons, between these and the already delaminated cells, as well as among cells in the forming ganglion.

**Conclusions:**

This new imaging assay provides a powerful method to analyze directly development of placode-derived sensory neurons and subsequent ganglia formation for the first time in amniotes. Viewing placode development in a head cross-section provides a vantage point from which it is possible to study comprehensive events in placode formation, from differentiation, cell ingression to ganglion assembly. Understanding how placodal neurons form may reveal a new mechanism of neurogenesis distinct from that in the central nervous system and provide new insight into how cells acquire motility from a stationary epithelial cell type.

## Background

Most studies on generation of neurons in vertebrates have focused on the central nervous system, which is composed of the brain and the spinal cord. The peripheral nervous system contains sensory neurons whose neurogenesis has been much less explored. In the head, ectodermal placodes contribute to a majority of the peripheral neurons, including sensory neurons in the cranial ganglia of the trigeminal V, facial VII, glossopharyngeal IX, and vagal X nerves responsible for somatosensation, general visceral sensation, and gustation (reviewed in [1,2]). They also give rise to essential components of the paired sense organs (lens, inner ear, and olfactory epithelium). Placodes are derived from discrete, usually thickened, regions of the embryonic head ectoderm in both sides of the forming neural tube. Placodes that form cranial ganglia are exclusively fated to become sensory neurons in the distal portions of the ganglia [3,4]. The generation of placodal neurons from the embryonic head epithelium is an intriguing process that differs from neurogenesis in the central nervous system (CNS), which takes place entirely within the neural tube.

Ectodermal placodes together with another embryonic cell population, the neural crest, are responsible for formation of the entire peripheral nervous system in vertebrates [1,3]. Compared with neural crest, however, much less is known about the mechanisms that govern formation of placodes. Several signaling pathways, including Wnts and Fgfs, have been implicated in playing a role in induction and differentiation of trigeminal (V) and epibranchial (forming the VII, IX, and X nerves) placodes [2,5-7]. However, the dynamic cellular processes by which these placode-derived cells delaminate from the epithelial ectoderm, migrate and coalesce in the underlying mesenchyme to form sensory ganglia remain poorly understood.

Placode formation involves induction of placode-specific fates and often acquisition of thickened columnar morphology followed by two key steps: first, the birth of neuronal cells in the ectoderm and the second, their detachment and migration to the site of ganglion assembly. The first step likely involves intricately regulated patterns of mitoses to generate the proper number and position of placodal neurons in the ectoderm. Previous studies show that placodal cells differentiate as neurons in the ectoderm prior to their delamination, as evidenced by co-labeling of fluorescently tagged ingressing placodal cells by GFP or vital dye with pan-neuronal markers Islet-1, neuronal beta-III tubulin (TuJ1), and neurofilament [8,9], and on cell morphology [10], showing that by the time of delamination placodal cells are already neurons. Furthermore, neuronal markers are detected in some scattered individual cells within the placodal ectoderm prior to placodal cell ingression [4,8]. These findings suggest that neurogenesis takes place in the embryonic ectoderm, and that placodal cells delaminate as neuronal cell types. Whether ectoderm cells divide symmetrically or asymmetrically to give rise to placode and non-placode cell types and where they divide along the apical-basal sides of the ectoderm remains largely unknown. Such differential modes of division (symmetric versus asymmetric) have been implicated in cell fate decisions in the CNS [11,12]. Phospho-histone H3 staining suggests that mitosis occurs apically in epibranchial placodes, and sectioned tissue suggests that placodes are pseudostratified in the embryonic epithelium [10]; however, this has yet to be analyzed directly by live-cell imaging.

The second step requires that placodal neurons make a transition from an epithelial to a mesenchymal like state in order to detach from the ectoderm. This process is often referred to as either ingression or delamination. However, placodal cells appear to undergo a different process of delamination than that of neural crest that undergo an epithelial-to-mesenchymal transition (EMT). For example, placode cells do not express typical EMT genes (i.e. Snail2 and the GTPase RhoB [13,14]) and express neuronal markers at the time of delamination [8,10]. How placodal cells change morphology to detach and acquire motility and how they migrate from the epithelial ectoderm remains elusive. Therefore, analyzing these cells in real time promises to reveal new insights into placodal cell behavior during development.

To date, in vivo imaging of placodal cell ingression in whole intact embryos remains optically difficult, largely due to the insufficient z-axis resolution in three-dimensional fluorescence microscopy. In particular, poor cellular resolution along the z-axis prevents clear delineation of cells in the surface epithelium from those that have migrated deep inside the head. To circumvent these issues, we have developed an imaging assay using cranial slice culture to monitor placodal cell behavior in real time at a single cell resolution. This novel imaging assay is based on adaptation of a long-term embryo slice culture imaging system previously established for chick spinal cord [12]. Similar slice imaging assays have been powerful in elucidating the dynamics of migrating and dividing neuronal cells in the mammalian cortex [15-17]. Here, we show that this imaging assay is highly effective for capturing different events in placode formation, ranging from cell proliferation, delamination and migration to assembly of these cells into ganglia. We focus on trigeminal placode formation as these form the largest of the cranial sensory ganglia, and are the first to form, making them highly tractable for imaging early placode formation.

## Results and discussion

### Development of cranial slice imaging system using the chick embryo

To image directly placodal cell delamination from the epithelial ectoderm and subsequent cell migration into the underlying mesenchyme, we sought to develop a method to visualize these tissues in a section of the embryonic head using three-dimensional fluorescence microscopy. Using 3-D wide-field deconvolution microscopy on embryonic slices, Wilcock et al. achieved the first time-lapse imaging of chick neurogenesis in the developing spinal cord at single cell resolution for >36 hours of imaging. Cell survival and cell cycle times in such cultures were similar to those in the intact embryo, demonstrating that the slice culture technique maintained the tissue and can be used to capture normal cell behavior. The general scheme of this imaging assay involves four main steps: first, introduction of fluorescent reporter constructs in the chick embryo by in ovo electroporation, second, surgical explant of a slice section from the embryo head, third, embedding slice onto a glass-bottom dish (see Methods) to image sample on an inverted microscope, and fourth, time-lapse imaging.

As chick embryos can be easily transfected by in ovo electroporation with a fluorescent reporter construct in a tissue-specific manner [18], this method was used to label future trigeminal placodal cells. To this end, the surface ectoderm overlying the embryonic head at the axial levels between the telencephalon and the otic vesicles, was transfected with reporter constructs (see Methods). This labels the targeted ectoderm, including the placodal ectoderm-derived cells that later delaminate into the mesenchyme [8,19]. The combination of a nuclear marker with either a cytoplasmic or membrane fluorescent reporter was found to work optimally for labelling and imaging individual placodal cells; the nuclear marker facilitated identification of individual cells while cytoplasmic or membrane markers revealed cell shape. Using histone H2B-RFP which labels the chromatin together with membrane or cytoplasmic GFP constructs to co-label the placodal ectoderm, we were able to capture both mitosis as well as changes to cell morphology over time.

**Figure 1:**
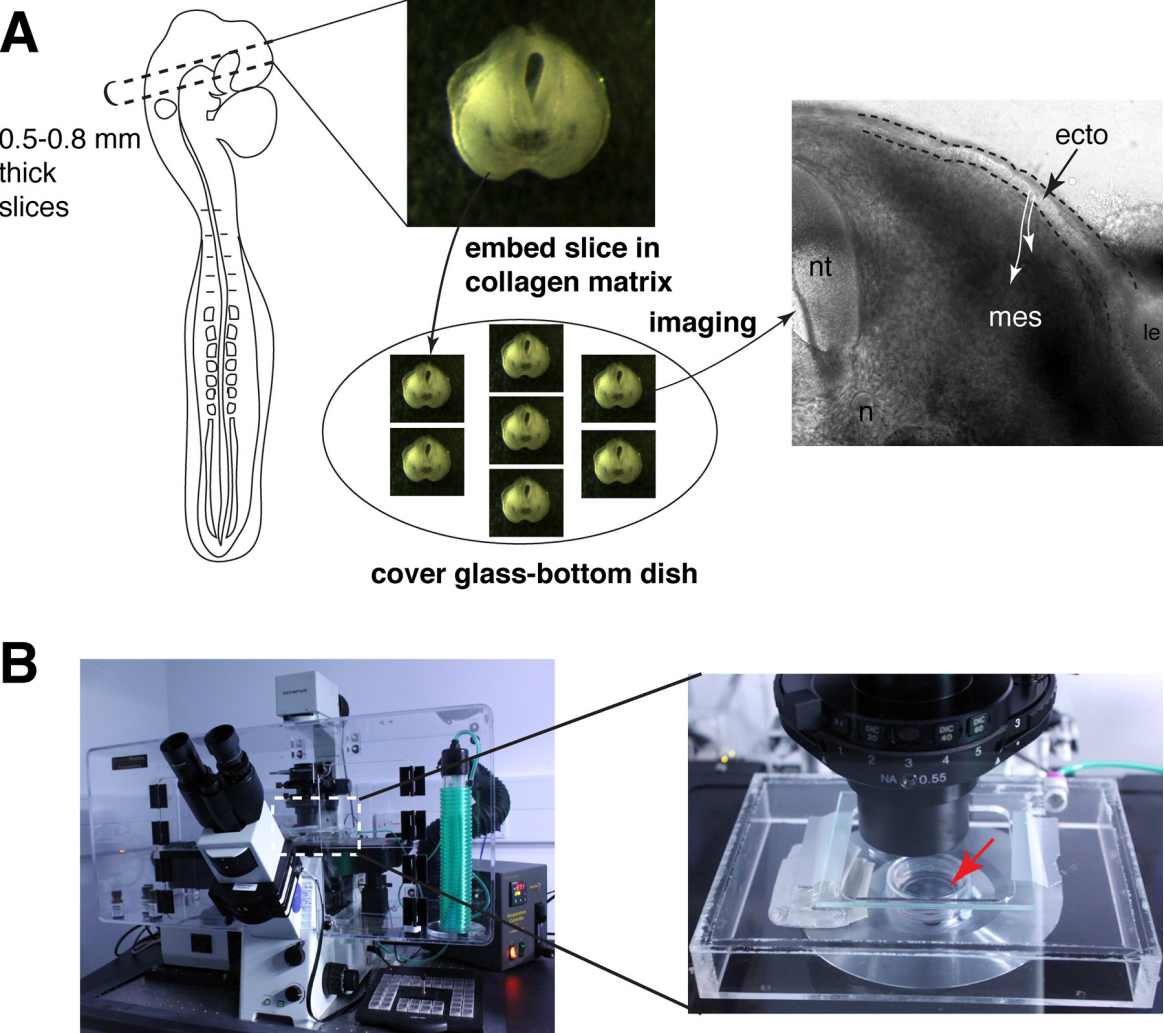
**Setup of the cranial slice imaging system**. (A) Schematic showing steps to making a cranial slice. A slice of a 0.5-0.8 mm thickness is excised at the dotted lines through the trigeminal region of the embryo shown by the cartoon which is then embedded in a collagen matrix on the cover glass-bottom dish. A representative slice (right) showing high cellular resolution when tissue is in focus, where ectoderm (ecto), mesenchyme (mes), neural tube (nt), notochord (n), and lens (le) are clearly delineated. White arrows show direction of placodal cell migration into mesenchyme. (B) DeltaVision Core microscope workstation with a built-in incubator chamber (left) and magnified view of the cover glass-bottom dish with several collagen cultures (red arrow) on the microscope stage with a carbon dioxide/air mix input source.

Electroporated embryos were screened in ovo for strong fluorescent reporter expression, and selected embryos were subsequently dissected out. To prepare embryos for slice explants, they were arranged and pinned around their extraembryonic tissues on clear sylgard dishes (see Methods). A surgical microknife was used to make two parallel sections across the head as shown in the Figure 1A. Slices could be made at all stages of trigeminal gangliogenesis from the start of ingression at stage 12 continuing to post-condensation of the ganglion at stage 18. At most two slices could be made per embryo, each ~ 500-800 um. The most important consideration for making slices for high quality imaging is to ensure that the side of the slice to be imaged is completely flush with the bottom cover glass so that cells are within the working distance of the lens. To avoid imaging cells at the cut edge, image stacks were commenced at least 2-3 cell layers within the tissue.

Cranial slices were maintained for ~ 12 hours at 38°C incubation with 5% CO_2 _using the same collagen based culture conditions in neurobasal media as used for spinal cord slices [12] (see Methods). Bright field microscopy was used to monitor the growth of the slice and the integrity of the epithelial ectoderm and mesenchyme layer (see representative bright field images of slices in Figure 1A). Some variability in viability and tissue integrity was observed among the slices, even in those from the same embryo and so is likely due to tissue handling rather than procedures applied to the donor embryo. To monitor viability and tissue growth a bright field image of the middle image of each z-stack was taken. This revealed that the surface ectoderm and the underlying mesenchyme were more prone to abnormal outgrowth and to loss of tissue integrity than the spinal cord, which is amenable to long term imaging over 36 hours [12]. The culture period of these cranial slices was therefore restricted to less than 24 hours, during which consistent normal growth was found for 3 to 8 hours and sometimes up to 12 hours (see additional file 1: Movie 1 showing a slice with normal growth over 8 hours). Movies of slices were only analysed if they showed preservation of tissue integrity and normal growth throughout the selected time period up to 12 h.

Time-lapse imaging was performed on a DeltaVision core widefield fluorescence microscope (Applied Precision), which has precise x-y stage control for automated imaging of multiple samples and z-stage control for collecting z-stacks (Figure 1B). For each experiment up to seven independent time-lapse movies were made, one for each slice positioned on the glass-bottom dish. Slices were imaged in a humidified chamber maintained at 38°C and buffered with 5% CO_2_/95% air mix (Figure 1B). Using wide-field fluorescence imaging has the advantage of faster image capture as it takes an entire image plane at once instead of a single pixel collection as is the case with conventional spot-scanning confocal microscopy [20]. This minimizes photobleaching and phototoxicity for 3-D imaging. By using an oil immersion 20 × objective (Olympus Plan S APO, NA 0.85), our cell labeling technique created high local fluorescent signals that could be detected across a broad field of cells. For each time-lapse recording, 17 - 50 optical sections were collected spaced by 3-5 um at 3 minute intervals for up to 20 hours. Each optical section had an exposure time of 5-50 miliseconds, with an actual total scan time of 5-20 seconds per stack per time point for 1-3 channels collection. The chosen optical section spacing and time-lapse intervals provided sufficient spatial and temporal resolution to capture dynamic processes of placodal cell projections, cell division, delamination and migration which occur over a time scale of several minutes to an hour.

### Time-lapse imaging reveals randomly oriented mitoses along apical-basal axis and cell division subsequent to ingression of trigeminal placode cells

This imaging assay was applied to visualize mitosis in the living trigeminal ophthalmic placodal tissue, which gives rise to the first placodes that later form the ophthalmic lobe of the trigeminal ganglion. Imaging was carried out at stages 13-14 when abundant placodal ingression has begun. Target tissue was double-labelled with a membrane localizing GFP reporter to visualize cell morphology and fine processes and a histone H2B-RFP reporter to mark cell nuclei and follow the progression of mitosis. These constructs were introduced into the trigeminal ectoderm by in ovo electroporation, which transfects ectodermal cells that constitute a heterogeneous population of non-placode and placode cells. Placode cells form in an apparently salt-and-pepper manner in the trigeminal ectoderm based on expression of placodal markers in discrete spots, such as Brn-3a [21], Neurogenins 1 and 2 [4], Robo2 [8], and Glypican-1 [22] as well as Notch pathway genes [23]. However, all labeled cells that delaminate from the ectoderm are of placodal origin. Due to a lack of chick placode-specific reporter transgene, this is currently the most effective method for labeling placodal cells. Although placode cannot be distinguished from non-placode cells in the ectoderm, we were able to retrogradely track delaminated placodal cells to their original positions in the ectoderm and thus identify individual placode precursor cells in our time-lapse data sets.

**Figure 2:**
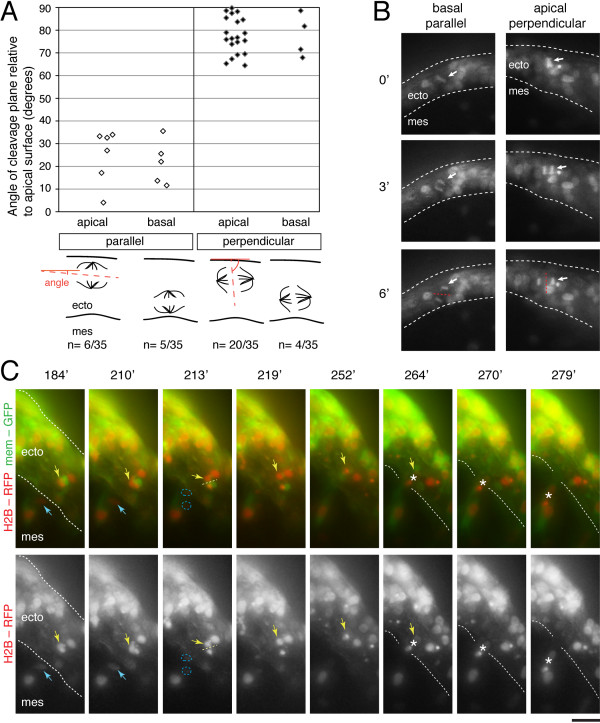
**Time-lapse imaging of mitoses in the trigeminal placodal ectoderm and ingressed cells**. (A) (Top) Dot plot showing parallel and perpendicular cleavage planes of dividing cells in both apical and basal sides of the placodal ectoderm. (Bottom) Schematic showing the measurement of the cleavage plane orientation based on the angle made by the cleavage line at anaphase relative to the apical surface. (B) Static images from projections of 24 um z-stacks showing two differently orientated mitoses in 6 minutes of real time between metaphase and anaphase: basal parallel and the apical perpendicular, also shown in additional files 2 and 3: movies 2 and 3. They were taken at 3 minute intervals using a 20 × objective. Cleavage plane is denoted by a red dotted line and dividing cell by a white arrow. (C) Image sequence over 95 minutes of development of a 2D projection of a 45 um z-stack taken at 3 minute intervals using a 20 × objective. H2B-RFP showing nuclei and membrane-GFP showing cell morphology. Dotted lines delineate the ectoderm layer; yellow arrow points to a dividing cell just before ingression (asterisk) with its cleavage plane demarcated by yellow dotted lines; blue arrow points to a cell that divides after ingression. Note that the delaminating cell (yellow arrow and asterisk) moves from within the ectoderm to exit this tissue. See additional files 4 and 5: movies 4 and 5 for the entire sequence. Scale bar: 10 um. ecto, ectoderm; mes, mesenchyme.

Using this cranial slice imaging assay many dividing cells were apparent in the placodal ectoderm and some were also observed in the mesenchyme. This prevalent detection of mitoses provides confidence in the viability and integrity of the cranial slices imaged. We found that the trigeminal placodal ectoderm contains mitotic cells all along its apical-basal axis without an apparent restriction to either side of the epithelium (Figure 2A). This is different from the apically restricted mitoses in the CNS, where the process of inter-kinetic nuclear migration, characteristic of pseudostratified epithelia involves nuclei moving between apical and basal surfaces as the cell progresses through the cell cycle (reviewed in [24]). Cell divisions that took place only in the x-y plane of the cranial slice were analysed as it is difficult to clearly visualize daughter cells that move deep into the slice. Mitoses were classified in the ectoderm into two general groups based on their cleavage planes: "parallel" means a cell that divides at ~ parallel (less than 45 degrees) plane relative to apical surface of ectoderm and "perpendicular" means a cell that divides along the length of the ectodermal sheet with a cleavage plane (greater than 45 degrees) (Figure 2A, B and additional files 2 and 3: movies 2 and 3). Cleavage planes as demarcated by dotted lines at anaphase (see schematic in Figure 2A) were measured as described in the Methods. We found that more than 65% of the divisions in the ectoderm had a perpendicular cleavage (above 60 degrees, n = 24/35 cells over 5 independent slices) (Figure 2A). Cells dividing with a perpendicular cleavage generate new cells within the plane of the ectoderm. These divisions may account for the expansion of the epithelial sheet to accommodate the growth of the embryonic head. There were less parallel cleavages (less than 40 degrees, n = 11/35). Although far more perpendicular cleavage divisions occurred near or at the apical side (n = 20/24, 83%) of the ectoderm than at the basal side (n = 4/24, 17%), there was no apparent difference in localization of nuclei along the apical-basal axis for the parallel cleavage divisions (n = 6/11 apical versus n = 5/11 basal) (Figure 2A). Some mitotic cells in the ectoderm exhibited rotation of their metaphase plate (indicating spindle rotation) prior to anaphase (see additional file 2: Movie 2).

In the time course of these time-lapses, we were generally unable to capture delamination following observation of mitosis. This is likely due to the long temporal separation of these two events and also in some cases the fact that the placodal cell nucleus in the ectoderm went out of focus in the z-axis. However, a few instances in which delamination followed soon after mitosis were observed (Figure 2C, additional files 4 and 5: movies 4 and 5) and that these ingressed ophthalmic placodal cells can undergo another round of cell division in the mesenchyme (Figure 2C, additional files 4 and 5: movies 4 and 5). This is consistent with previous static analyses showing exceptional cases of placodal cells in S-phase in the mesenchyme using a short pulse thymidine analog (BrdU) labeling [8]. Based on expression of neuronal markers TuJ1 and Islet1, ingressed cells are already neurons [9,25]. Other reports have suggested that the ingressed ophthalmic placodal cells were post-mitotic based on neuronal marker expression, absence of mitotic marker phospho-histone H3, and early cell cycle exit based on tritiated thymidine or BrdU labeling [4,9]. Our time-lapse imaging data largely support these studies, but also reveal that not all ingressed ophthalmic placodal cells are post-mitotic. Since expression of mitotic markers are short-lived and transient as mitosis is estimated to last about 30 minutes in the chick embryo [12], it is possible that the few instances of cell division in ingressed placodal cells went undetected in those studies. Time-lapse imaging of this process thus allows capture of mitoses more accurately, allowing direct tracking of individual placode cells through ingression independent of marker analysis. Furthermore, expression of pan-neuronal marker TuJ1 in trigeminal placodal neurons is found at or near their terminal division [26], consistent with the possibility that cells expressing these neuronal markers are not definitively post-mitotic.

Taken together, the findings show that our imaging assay provides an effective experimental platform to investigate how regulation of cell cycle is related to placodal differentiation and delamination.

### Time-lapse imaging of trigeminal placodal cell ingression and the ganglionic anlage reveals focal delamination points and dynamic intercellular contacts

**Figure 3:**
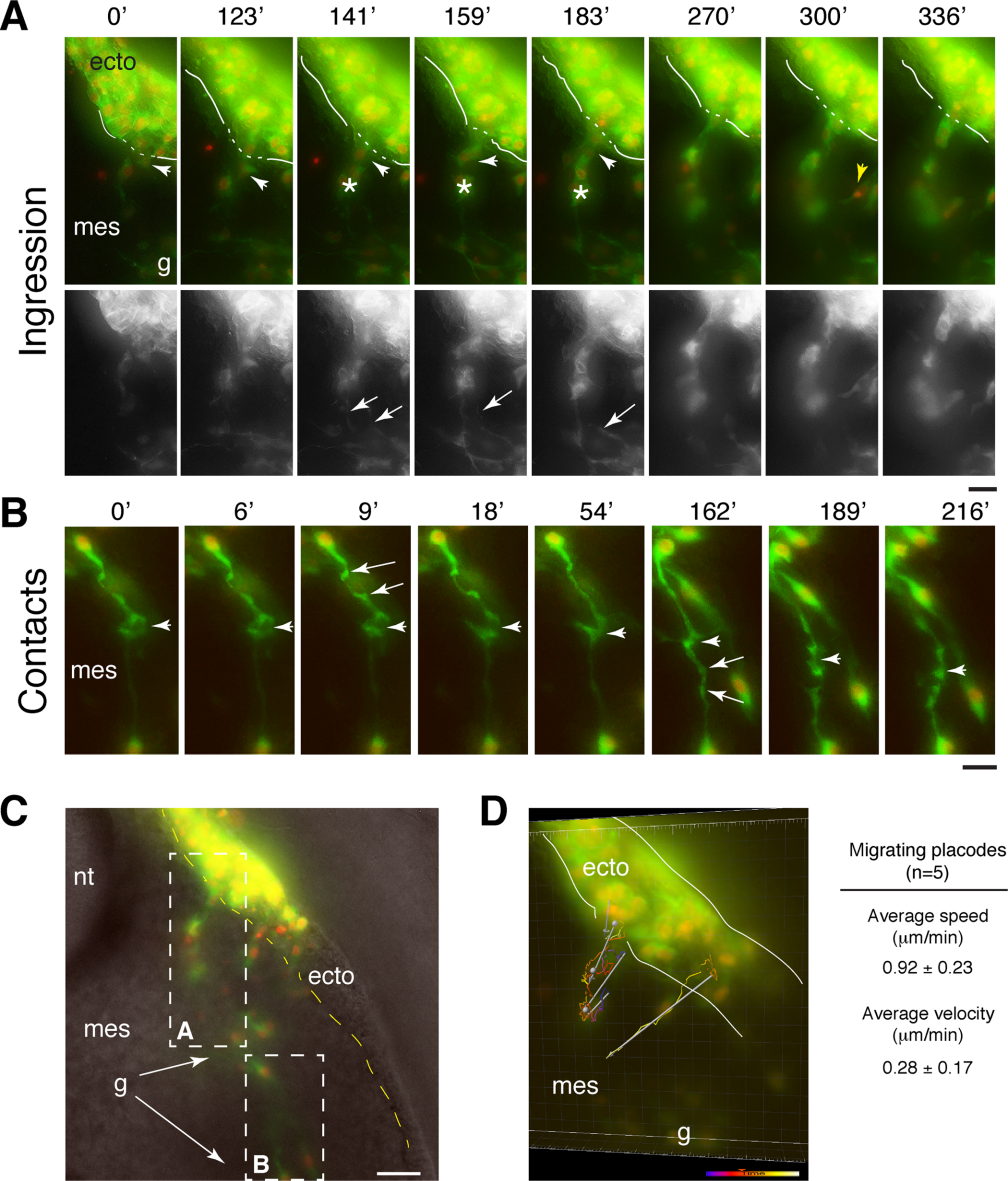
**Time-lapse imaging of ingression and cellular contacts by trigeminal placodal neurons in the mesenchyme**. (A) Sequence of images showing actively ingressing placodal cells labeled by membrane GFP in green (also shown separately in bottom gray panels) and nuclear H2B-RFP in red. Ingressing cells delaminate as a short string of cells (arrowhead) through a common exit point on the basal side of the epithelium to enter the mesenchyme (mes). Solid white lines delineate the basal surface of ectoderm with dotted lines marking the site of placodal ejection. Newly ingressing cells (asterisk) remain in close contact with one another while also making contacts (arrows in bottom panels) with the placodal cells which have already reached the ganglionic anlage (g) and have begun to assemble into a ganglion mass. The full sequence of images are shown as time-lapse videos in additional files 7 and 8. (B) Highly dynamic axonal contacts are observed between placodal cells in the ganglionic anlage. Changes to axonal morphology, including irregular cytoplasmic protrusions, were found (arrows) and growth cones collapse at time of connection (arrowhead). Movie is shown in additional file 9. (C) Fluorescence images are superimposed on the bright-field image at one time point showing the view of the entire cranial slice that can be broken down to show different steps of placode development: ingression as shown in A and cell-cell interactions as shown in B. Yellow dotted line marks the edge of the ectoderm. (D) Image showing a volume rendering of the cranial slice over time using Imaris. Trajectories of migrating placodal cells are shown by colored lines that change from blue to red to white with progressing time, which was used to calculate average speed (n = 5). Displacements of placodal cell movement are indicated by the solid white arrows, which was used to calculate average velocity (n = 5). Scale bar: 10 um. ecto, ectoderm; mes, mesenchyme; nt, neural tube; g, ganglionic anlage.

Imaging at stages 14-15 during the peak period of ingression [3] was found to be optimal for capturing trigeminal placodal cells as they delaminate. The success rate for monitoring ingression is much lower at earlier stages (11-12) as fewer cells are undergoing this process. In addition, fluorescently labeled ingressing or already ingressed cells at stage 14 can be used as landmarks to indicate where cells are likely to delaminate from the ectoderm. We co-labeled the trigeminal ectoderm by in ovo electroporation using the same reporter constructs as above. We used membrane GFP revealing cell morphology and processes, and H2B-RFP marking nuclei. Imaging cranial slices revealed some novel aspects of the delamination of trigeminal ophthalmic placodal cells (Figure 3A and see additional files 6, 7 and 8: movies 6, 7 and 8). Previous static analyses suggest that trigeminal placodal cells delaminate from numerous scattered foci comprised of thickenings or spurs of cells [3,9]. Interestingly, our time-lapse imaging revealed that the delaminating cells begin the process of ingression earlier in time, moving first within the ectoderm (additional file 6: movie 6), and then exiting as individual or strings of cells through common openings on the basal side of the epithelium (Figure 3A) (n = 3 independent slices). In fixed tissue sections, ingressing placodal cells that migrate out as short strings of cells may appear like these numerous scattered foci (thickenings or spurs) as described previously [3,9]. These common sites of placodal exit likely involve a breakdown of the basal lamina, inferred from static analyses of epibranchial placodes [10], fine resolution ultrastructure studies of mouse trigeminal placode [27] and ultrathin sections of delaminating chick otic placodes [28].

The abundant cell-cell contacts among placodal cells themselves throughout placodal development are striking. We observed intimate contacts of cellular processes between placodal cells at the time of ingression, between newly delaminated placodal cells with those that are already positioned in the ganglionic anlage, and placodal cells within the ganglionic anlage (Figure 3A-C). There appear to be interactions between newly ingressing cells with those already in the mesenchyme as these cells are constantly projecting processes towards each other (Figure 3A, additional files 7 and 8: movies 7 and 8). As cells ingress, we also notice that they not only send processes in the direction of their movement but also back towards the ectoderm from which more cells follow (Figure 3A, additional files 7 and 8: movies 7 and 8). Based on their bipolar morphology with two processes at opposite ends of the cell body, the placodal cells that have left the ectoderm appear neuronal (Figure 3A) consistent with previous studies [8,9,25]. The newly ingressing placodal cells have two short processes and have the appearance of younger neurons than those already migrated which have long and more elaborate axonal processes (compare Figures 3A and 3B). Axonal morphology and contacts between mature placodal neurons were also observed to be highly dynamic and contacts eventually collapsed at time of connection (Figure 3B and additional file 9: movie 9). Compared with neural crest cells, placodal cells do not migrate as far or as fast, with an average velocity of 0.28 +/- 0.17 um/min and an average speed of 0.92 +/- 0.23 um/min or 55 +/- 13.8 um/hour (Figure 3D), which is about one-third the average speed of neural crest migration into branchial arch 1 (158 +/- 2 um/hour) [29]. This is not too surprising as placodal cells, unlike neural crest, do not migrate extensive distances to reach their target sites and appear to form a cohesive group of cells as soon as they enter the mesenchyme.

### A comprehensive view of placodal development from origin in the ectoderm to final localization within the ganglion as viewed in transverse section

**Figure 4:**
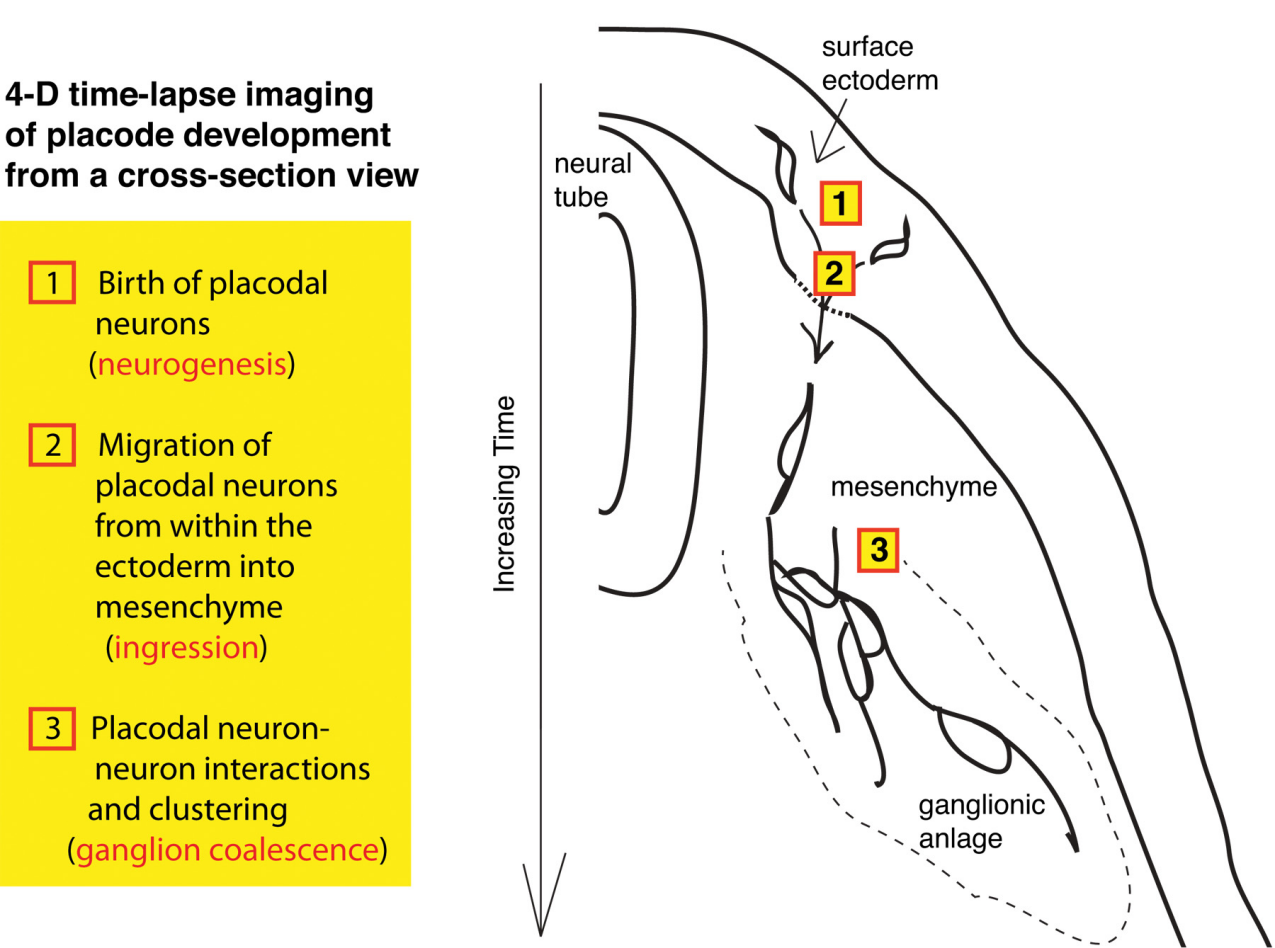
**Embryonic head cross-section provides a comprehensive view of placodal development**. Time-lapse imaging of a cranial slice allows direct visualization of placode formation and morphogenesis through time in one field of view, from (1) the birth of placodal neurons in the ectoderm, (2) delamination of placodal neurons, and (3) maturation and aggregation of placodal neurons into ganglia in the mesenchyme. Imaging data reveal dynamic changes in placodal cell processes and morphology throughout gangliogenesis.

The formation of placode-derived sensory neurons occurs continuously over a relatively long period of time. Ingression of trigeminal placodal cells from the ectoderm persists for about 1.5-2 days of normal chick development (~ stages 12-21), although the peak period is during stages 14-16 [3]. As it is a continuous process, the dynamics of placodal cell ingression and ganglion assembly can be captured by short time-lapses over a few hours within a rather flexible time window of embryonic development. Live cell imaging on cranial slices encompassing the transverse-section view of the embryonic head through the ganglionic anlage lays out the full sequence of events starting from formation of placodes in the ectoderm, then delamination of placodal cells, to interactions among placodal cells as they assemble into ganglia in the underlying mesenchyme (Figure 3C and 4). At any one time point, therefore, during active gangliogenesis, all the key steps to placodal development can be visualized in one field of view.

## Conclusions

The generation of new placodal cells from the ectoderm and incorporation into the ganglionic anlage is a continuous process that occurs over about two days of normal chick development. Imaging short time windows of about 3 to 8 hours using this cranial slice imaging assay was sufficient to capture dynamic changes in placodal cell morphology during ingression, migration, and ganglion assembly as well as to monitor mitoses. Interestingly, the time-lapse imaging data revealed that trigeminal placodal cells begin their ingression early on from within the ectoderm. This extends previous observations made in fixed tissues, which identified spurs or thickenings on the basal epithelial surface and concluded that ingression commenced at these points. We found that ingressing cells do not leave the ectoderm through individual exit points, but instead move into the mesenchyme via exit points used by placodal cells that ingressed before them. The data suggest that there are discrete common openings in the ectoderm from which placodal cells leave. The imaging data provide clear evidence of constant and abundant contacts between placodal processes throughout gangliogenesis. They show that mitoses in the trigeminal placodal ectoderm are not restricted to either apical or basal sides and have different cleavage orientations. The data also suggest that many, but not all, ingressed trigeminal placodal cells are post-mitotic. Taken together, the data shed new light on the early migration of placodal cells from within the ectoderm and their highly interactive processes throughout their development, which were not apparent in previous static analyses.

In summary, we describe a chick cranial slice imaging assay which is highly effective for visualizing and analyzing sensory placode development. Combination of this live placodal imaging assay with molecular perturbations involving gain and loss of gene function will allow study of the spatiotemporal dynamics of the mechanisms that drive formation of the placode-derived cranial sensory ganglia, which until now have remained limited to static analyses on fixed whole mount and sectioned embryos. Study of the regulation of how these cells delaminate may also help to determine whether this is similar to the classical epithelial-to-mesenchymal transition and would further address the long-standing question of how cells acquire motility from a stationary epithelial cell type. Detailed investigation into how placodal neurons form would contribute significantly to our current understanding of neurogenesis. As inter-kinetic nuclear migration is lacking, the way in which placodal cells become neurons may reveal a new mechanism distinct from that operating in the CNS.

## Methods

### Chick embryo manipulations

Fertilized chicken (Gallus gallus domesticus) eggs were obtained from Winter Egg Farm (Hertfordshire - Royston SG8 7RF, UK) and incubated at 38°C to the desired stages. Reporter constructs were transfected into the trigeminal ectoderm by in ovo electroporation at stages 9-10 (7-11 somites stages). Plasmids were injected overlying the presumptive trigeminal placodal ectoderm at the approximate axial level between the posterior forebrain and anterior hindbrain. To electroporate, platinum electrodes were placed vertically across the chick embryo delivering current pulses of 5 - 8 V in 50 ms at 100 ms intervals as described [8]. Targeting DNA to the ectoderm resulted in transfection of the trigeminal placodes in the ectoderm and their derived cells. The operated eggs were sealed and incubated at 38°C for ~ 16-24 hours to reach stages 13-14, ~ 24-36 hours to reach stages 15-16, and ~ 40-48 hours to reach stages 17-18. Electroporated eggs were screened and selected for strong GFP/RFP expression and processed for slice culture and imaging. Reporter constructs used were: cytoplasmic GFP (cytopcig) [8], membrane GFP (pCAβ-IRES-myristoylated GFP), and nuclear RFP (pcig-H2B-RFP) (gift of T. Sauka-Spengler), which were injected at an initial concentration of 2-4 ug/ul.

### Cranial slice preparation and culture

Slices were prepared as described in Results and Discussion. Transfected embryos that were selected for strong fluorescence expression were collected at the desired developmental stages between 12-18 for imaging. Transverse sections were surgically excised using a microknife with a 15 degrees blade slant (Altomed, A10102) from the midbrain level of the head ectoderm, targeting the trigeminal ophthalmic region in the dorsal half (Figure 1A). One to two cranial slices were made per embryo. For making slices from embryos older than stage 12 when the head has become mostly or completely detached from the extraembryonic tissue, we also added two pins to the head outside the trigeminal ectoderm, one either in the telencephalon (before head turning) or in the midbrain ventricle (after head turning), plus a second pin through the otic vesicle. Each slice was embedded in collagen at a final concentration of 2.3 mg/mL (Type I rat tail, BD Biosciences, 354236) including L-15 medium (Gibco) with final concentrations of 0.02% acetic acid and ~0.25-0.35% of sodium bicarbonate to polymerize the collagen. Collagen-embedded slices were made on 35 mm WillCo glass-bottom dishes with 22 mm diameter and 0.17 mm thick glass which fit at most seven cranial slices. Each slice was oriented and positioned as flush as possible to the bottom glass in collagen prior to polymerization, one at a time. After all slices have been embedded and collagen has polymerized, culture media (neurobasal media from Gibco without phenol red with B-27 supplement (Gibco), L-glutamine (Gibco), and gentamycin (Invitrogen)) was gently placed over the collagen to fill half the dish volume. Slice cultures were equilibrated to developmental temperature at 38°C with 5% CO_2 _level for at least one half hour prior to imaging.

### Imaging chamber preparation

Prior to imaging, generally at least one day in advance of experiment, the cover glass base of the imaging chamber was treated and coated with poly-L-lysine (Sigma-Aldrich, P8920). Poly-L-lysine was incubated on glass for 15 minutes or longer up to one hour at room temperature, and was removed followed by 4-6 quick rinses with double-distilled or sterilized water. Excess leftover polymer can be toxic to the tissue. The cover glass was left to dry at room temperature over several days, in the 38°C incubator overnight, or for a quick dry by low power microwave heating at 2 minutes interval. A completely dried cover glass is necessary for setting collagen on the glass for slice preparation.

### 4D widefield fluorescence imaging

Cranial slices were imaged on a DeltaVision Core widefield fluorescence system (Applied Precision, Issaquah, Washington) as described in Results and Discussion. The microscope system uses an Olympus inverted microscope with a xenon lamp as the light excitation source and a high-speed cooled CCD camera (Photometrics CoolSnap HQ2). Slices were maintained at 38°C within a Solent environmental chamber. The DeltaVision acquisition software Resolve3D was used to control image acquisition.

### Cell tracking and image analysis

We used SoftWoRx (Applied Precision) for analyzing and projecting 3D stacks into 2D images. To measure speed and velocity of placodal cell migration in 3D volume, we performed 4D tracking with Imaris (Bitplane) by modeling moving placodal nuclei with spot objects using the ImarisTrack module with manual corrections and Imaris MeasurementPro for calculations of the tracks. The orientation of cleavage planes with respect to apical surface was measured by the angle made between a line bisecting two sets of chromosomes separating at anaphase (see schematic in Figure 2A) and an approximate tangent line of the apical ectodermal surface in projected 2D images. The angle is taken between 0 to 90 degrees in absolute terms (either clockwise or counterclockwise) using the angle measurement function in ImageJ. All images were processed using Adobe Photoshop CS5 and made into video files using Adobe Premiere Pro CS5 or ImageJ. To eliminate out of focus light using wide-field microscopy, this mode of imaging is often combined with deconvolution processing. However, this is not effective using a low NA value (<1.0) lens, which we employed here to provide a greater field of view.

## Authors' contributions

CES conceived of the project with contribution of its design from KGS and RMD. CES performed and analyzed all the experiments. RMD provided expertise on the DeltaVision imaging system and slice culture techniques. CES and KGS wrote the manuscript. All authors have read and approved the final manuscript.

## Supplementary Material

Additional file 1 **Time-lapse movie of a representative cranial slice in bright-field**. The image sequence is taken from the middle slice of a z-stack over the duration of the time-lapse imaging (8.4 hours). For each z-stack, a bright-field image was taken at the middle slice as control to monitor integrity of tissue in parallel with the fluorescence imaging. Images were taken at 3-minute intervals at 20×, and shown at 6.5 fps. ecto, ectoderm; mes, mesenchyme; nt, neural tube. Click here for file

Additional file 2 **Time-lapse movie of a dividing cell labeled by H2B-RFP undergoing a parallel cleavage in the basal side of the placodal ectoderm**. Metaphase plate rotates prior to division (arrow). 72 minutes in real time taken at 3-minute intervals; 24 um z-stack; 20×; 6.5 fps. Click here for file

Additional file 3 **Time-lapse movie of a dividing cell labeled by H2B-RFP undergoing a perpendicular cleavage in the apical side of the placodal ectoderm**. 60 minutes in real time taken at 3-minute intervals; 24 um z-stack; 20×; 6.5 fps. Click here for file

Additional file 4 **Time-lapse imaging of cell divisions (dotted lines around cells) in the trigeminal placodal ectoderm as labeled by nuclear H2B-RFP and membrane GFP**. Dotted white line demarcate basal edge of ectoderm. Yellow arrow points to a placodal cell that divides prior to ingression and a different placodal cell (blue arrow) undergoes division after it is already in the mesenchyme. Video covers 336 minutes of development in real time captured at 3-minute intervals; 45 um z-stack; 20×; 7.5 fps. Click here for file

Additional file 5 **Same as Movie 4 but showing only the H2B-RFP channel for clearer visualization of only nuclei, 6.5 fps**. Click here for file

Additional file 6 **Time-lapse imaging of the cranial slice in Figure **3C** showing only nuclear H2B-RFP labeled ectoderm and ectoderm-derived cells to follow movements of placodal cell bodies within the ectoderm and during delamination**. Two examples of placodal cells that move from within the ectoderm are each marked by a different colored dot (red and green) starting at a time point when the cell can initially be tracked. Tracking of the green cell begins after it has already begun migration. Some cells move in different directions before appearing to follow a path to the site of exit on the basal side of the epithelium (see red cell). 210 minutes in real time taken at 3-minute intervals; 45 um z-stack; 20×; 10 fps. Click here for file

Additional file 7 **Time-lapse imaging of actively ingressing cells labeled by membrane GFP and nuclear H2B-RFP from the placodal ectoderm**. Movie shows highly interacting placodal cells as they stream out of a discrete exit point on the basal side of the epithelium, forming contacts among themselves and with already ingressed ganglionic placodal neurons. Imaging shows 336 minutes of development captured at 3-minute intervals; 45 um z-stack; 20×; 6.5 fps. Click here for file

Additional file 8 **Same as Movie 6 but showing only the membrane GFP channel for clearer visualization of the cell morphology**. Click here for file

Additional file 9 **Time-lapse imaging of placodal neurons labeled by membrane GFP and nuclear H2B-RFP in the ganglionic anlage show intimate contacts by placodal processes**. Movie shows highly dynamic interactions at sites of axon-axon contacts which eventually make a connection at time of growth cone collapse. 336 minutes in real time taken at 3-minute intervals; 45 um z-stack; 20×; 6.5 fps. Click here for file
